# Supplementary material for: Mesoporous Organosilica Nanoparticles with Tetrasulphide Bond to Enhance Plasmid DNA Delivery
Source: Pharmaceutics. 2023 Mar 22;15(3):1013. doi: 10.3390/pharmaceutics15031013 (PMC10053670; doi:10.3390/pharmaceutics15031013)
Supplement: Supplementary file 1 [file pharmaceutics-15-01013-s001.zip › pharmaceutics-2198400-supplementary.pdf]

Communication

Mesoporous Organosilica Nanoparticles with Tetrasulphide Bond to Enhance Plasmid DNA Delivery

Yue Zhang [1], He Xian [1], Ekaterina Strounina [2], Kimberley S. Gunther [3], Matthew J. Sweet [3], Chen Chen [4], Chengzhong Yu [1,*] and Yue Wang [1,*]

[1] Australian Institute for Bioengineering and Nanotechnology, The University of Queensland, Brisbane, QLD 4072, Australia; yue.zhang18@uq.net.au (Y.Z.); h.xian@uq.net.au (H.X.)
[2] Centre for Advanced Imaging, The University of Queensland, Brisbane, QLD 4072, Australia; e.strounina@uq.edu.au
[3] Institute for Molecular Bioscience (IMB), IMB Centre for Inflammation and Disease Research, and Australian Infectious Diseases Research Centre, The University of Queensland, Brisbane, QLD 4072, Australia; k.gunther@uq.net.au (K.S.G.); m.sweet@imb.uq.edu.au (M.J.S.)
[4] School of Biomedical Sciences, Faculty of Medicine, The University of Queensland, Brisbane, QLD 4072, Australia; chen.chen@uq.edu.au
* Correspondence: c.yu@uq.edu.au (C.Y.); yue.wang1@uq.edu.au (Y.W.)

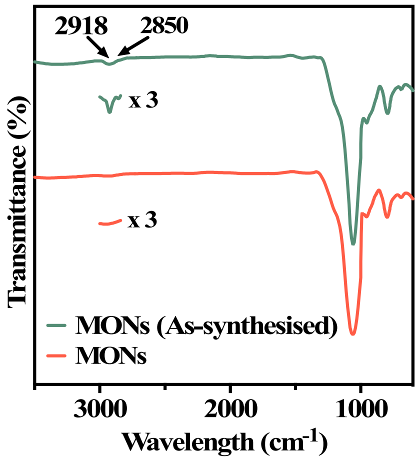

Figure S1. FTIR spectrum for MONs before and after acid extraction.

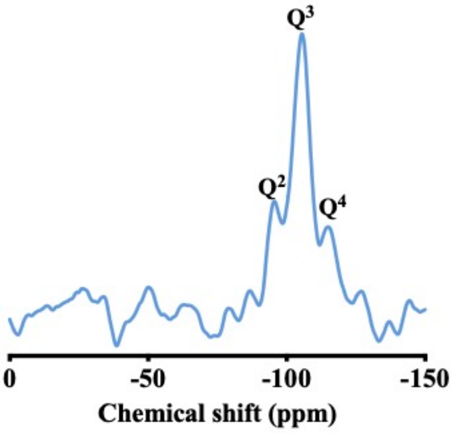

Figure S2. Solid-state ^{29}Si MAS NMR spectrum of MSNs.

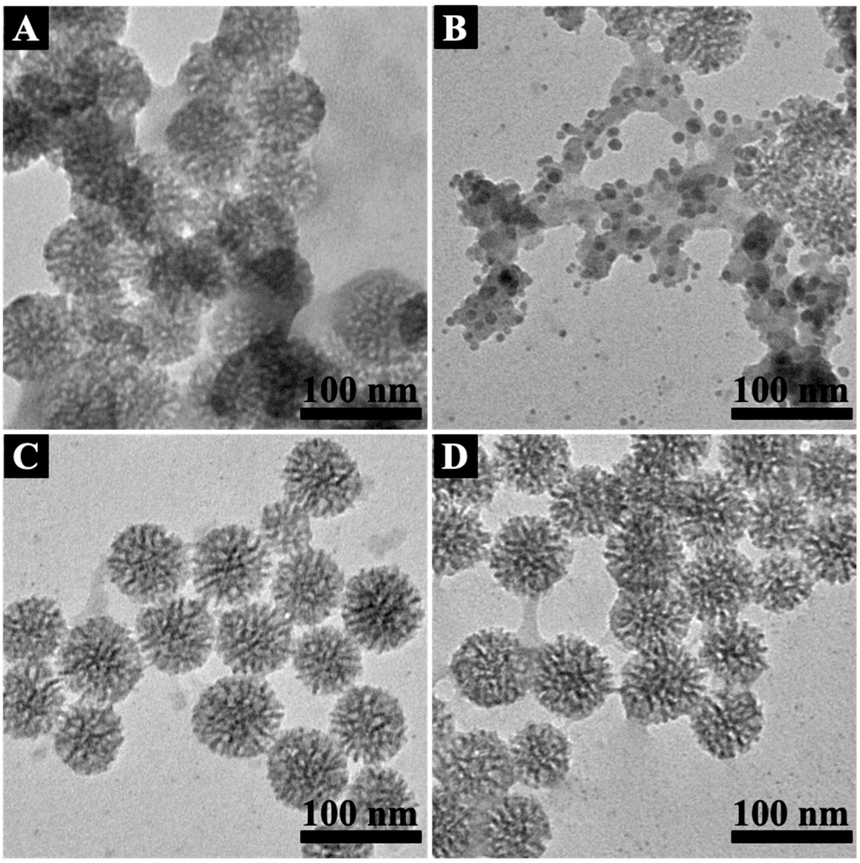

Figure S3. Degradation test of MONs (A-B) and MSNs (C-D) tested at 1 mM (A, C) and 10 mM (B, D) GSH solution for 48 h in the presence of serum.

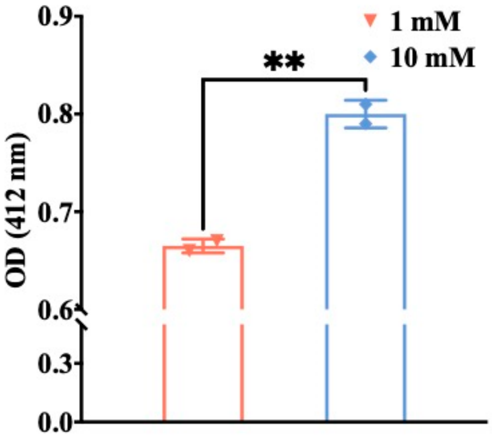

Figure S4. Relative quantification of -SH groups (OD 412 nm) after incubation of MONs in 1 mM and 10 mM GSH solution for 48 h in the presence of serum.

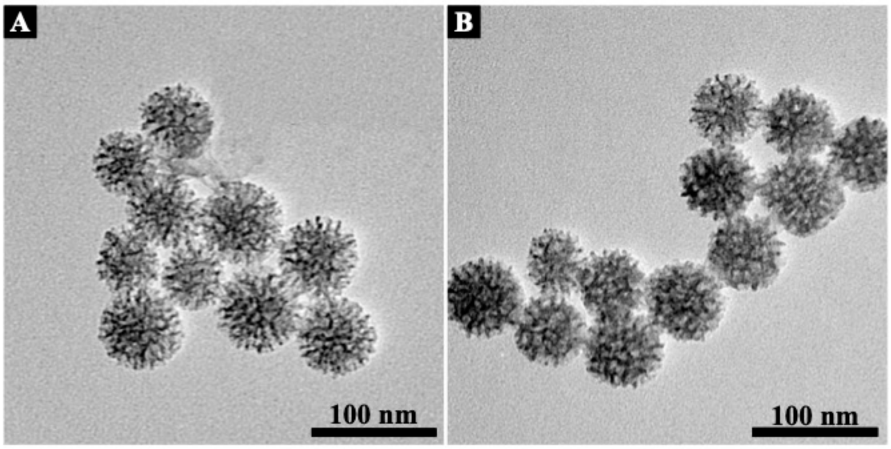

Figure S5. TEM images of PEI-MONs (A) and PEI-MSNs (B).

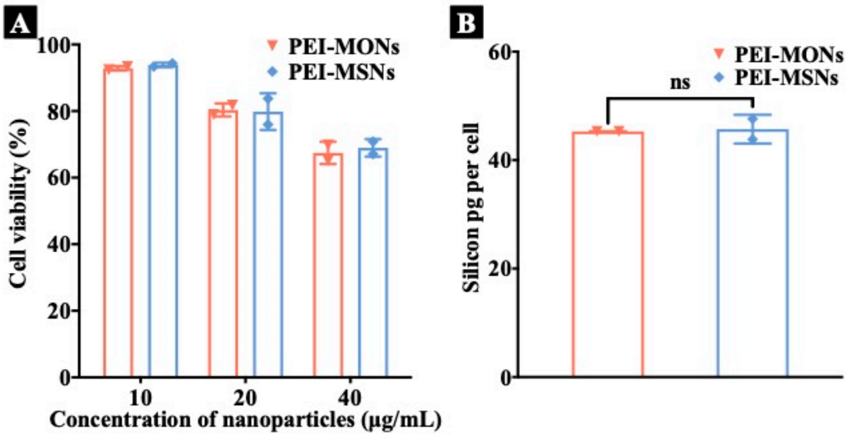

Figure S6. (A) Cell viability of DC2.4 cells after incubation with PEI-NPs for 24 h. (B) Cellular uptake of DC2.4 cells incubated with PEI-NPs for 4 h measured by ICP-OES.

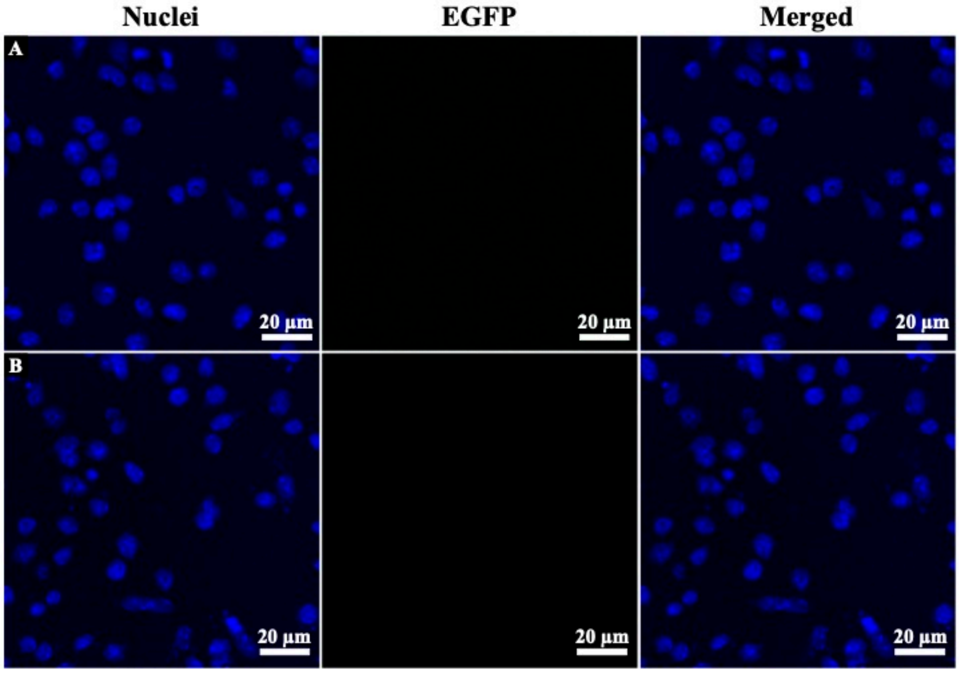

Figure S7. Confocal images of DC2.4 cells incubated with PBS (A) and bare EGFP pDNA (B).

Table S1. Composition properties of MONs and MSNs before and after PEI modification

Sample name	Nitrogen (%)	Carbon (%)	Sulphur (%)
MONs	0	8.5	12.6
MSNs	0	0	0
PEI-MONs	5.8	19.5	9.4
PEI-MSNs	5.7	12.3	0
